# Effect of Plant Essential Oil Formulations on *Bemisia tabaci* MEAM1 (Gennadius) and Its Parasitoid *Eretmocerus hayati* (Zolnerowich and Rose)

**DOI:** 10.3390/plants12244137

**Published:** 2023-12-12

**Authors:** Errol Hassan, Yasir Obaidoon, Md Munir Mostafiz, Lara Senior

**Affiliations:** 1 School of Agriculture and Food Sciences, University of Queensland, St. Lucia, QLD 4343, Australiayass.yess@gmail.com (Y.O.); 2 Agricultural Science and Technology Research Institute, Kyungpook National University, Daegu 41566, Republic of Korea; munirmostafiz12@gmail.com; 3 Department of Agriculture and Fisheries, Ecosciences Precinct, Dutton Park, QLD 4102, Australia

**Keywords:** silverleaf whitefly, parasitoid, essential oil formulations, environmentally friendly insecticides, sustainable agriculture

## Abstract

Silverleaf whitefly (SLW), *Bemisia tabaci* (Gennadius) (Hemiptera: Aleyrodidae), consists of genetically diverse species known to cause significant destruction in many crops around the world. Nowadays, synthetic insecticides are a key component in the management of this pest. However, they also come with disadvantages, such as environmental pollution, pest resistance and recurrence, and toxicity to pollinators and natural enemies. Essential oils from aromatic plants and biocontrol agents may provide a new and safe alternative to synthetic chemicals. In this study, we assessed the lethal impact of three new plant essential oil formulations (referred to as F1, F2, and F3) against the developmental stages of *B. tabaci* and its parasitoid *Eretmocerus hayati* (Zolnerowich and Rose) (Hymenoptera: Aphelinidae). The tested formulations consisted of combinations of mustard oil and different surfactants. The formulations were effective against the eggs and nymphal stages of *B. tabaci*. At the highest concentration assessed (1.23%), F1 was the most effective formulation against the eggs, resulting in 85% mortality, whereas F2 was most effective against the nymphs (92.5% and 88.3% mortality for the young and old nymphs, respectively). However, adult mortality rates were below 40% for all the tested formulations. The range of median lethal concentration (LC_50_) values was between 0.65 and 1.05% for *B. tabaci*. The side effects of the three formulations were assessed against *E. hayati*, treated as parasitized nymphs of *B. tabaci*. At the highest tested concentration (1.23%), F2 and F3 resulted in 80% and 70% mortality of the parasitoids, respectively (classified as moderately or slightly harmful according to the IOBC), whereas F1 resulted in 17.5% mortality. As F1 was effective against SLW with minimal effects on the parasitoid, it is the most suitable formulation of those tested for use in an integrated pest management (IPM) program targeting the younger life stages of *B. tabaci*.

## 1. Introduction

The silverleaf whitefly or SLW (MEAM1; formerly B-biotype)*, Bemisia tabaci* (Gennadius) (Hemiptera: Aleyrodidae), is one of the most serious agricultural insect pests worldwide in tropical and subtropical regions and in greenhouse production systems [1,2,3,4]. *B. tabaci* is a species complex of at least 40 cryptic species that are morphologically undistinguishable [1,2]. Among the species, the Middle East-Asia Minor 1 (also known as MEAM1) and the Mediterra-nean (MED, previously known as Q biotype) are the most damaging to various crop plants [4]. It has been recorded from more than 900 different plant species, including field crops, ornamentals, vegetables, and fruit crops [4,5]. The silverleaf whitefly damages plants by sucking the plant sap, causing stunting and reduced growth and yield [6]. This whitefly is also an important virus vector, transmitting more than 110 species of plant viruses, such as tomato yellow leaf curl virus (TYLCV) and tomato leaf curl virus (TLCV) [7,8].

Biological control methods against *B. tabaci* have gained increasing popularity in response to major problems caused by chemical control, including environmental pollution and the development of resistance against many commonly used pesticides [9]. The parasitoid *Eretmocerus hayati* (Zolnerowich and Rose) (Hymenoptera: Aphelinidae) is one of the 112 parasitoids of the *B. tabaci* species complex [10]. *E. hayati* is a primary, solitary parasitoid that oviposits externally on the dorsal surface of the nymphal host [11]. After eclosion, the first instar penetrates the abdomen of the host whitefly nymph and develops internally [12]. *E. hayati* parasitizes all *B. tabaci* nymphal instars except for the late fourth instar, with a preference for the first and second instars [12]. Originally from Pakistan, introductions of *E. hayati* as part of a biological control program proved to be successful for the management of *B. tabaci* in the USA [13]. The parasitoid was imported from western USA by CSIRO in 2002 for *B. tabaci* biological control in Australia, released from quarantine in 2004, and reported to be an efficient parasitoid for SLW control in vegetable production systems in Queensland [14]. It has been used successfully in Australia [15], USA [13], and Egypt [16].

The management of this whitefly has relied heavily on conventional insecticides, but these have significant drawbacks [5]. The silverleaf whitefly has developed resistance to many of the insecticides commonly used for its control [17,18,19,20,21]. Synthetic insecticides are also harmful to humans, the environment, and natural enemies [22,23]. For example, adults of *E. hayati* exposed to dried residue of dinotefuran (Starkle^®^) exhibited over 90% mortality two days post application [24]. In another study, bifenthrin (Talstar^®^), clothianidin (Shield^®^), and dinotefuran (Starkle^®^) showed a high toxicity (over 75% mortality) to *E. hayati* two days post-treatment [25]. The negative impacts of synthetic pesticides on the environment and natural enemies have prompted the development of new alternative pest control strategies [11,26]. Increasing attention to environmental safety has triggered interest in pest control approaches through eco-friendly plant-based pesticides [26,27,28].

Recently, a growing number of plant essential oils (EOs) have been tested against a wide range of arthropods pests [27,29,30]. EOs exhibited high effectiveness, multiple mechanisms of action, and low toxicity in non-target vertebrates [31,32,33,34]. Surprisingly, the number of commercial biopesticides based on EOs remains low, and this opens opportunities for their application in modern agriculture [35]. In the present study, we assessed the efficacy of formulations comprising mustard oil and surfactants. In earlier studies, mustard essential oil exhibited insecticidal activity against lepidopteran and storage pests [36,37]. In addition, surfactants have been shown to have insecticidal activity against the whitefly [38] and other insect pests such as stored product pests [39,40] and aphid species [41,42,43]. Surfactants may affect agricultural pests by disrupting the waxy layer of insect cuticles or by enhancing the toxic effect of essential oils [38]. However, although mustard oil and surfactants alone have been shown to present insecticidal activity against various insect pests, their effect on *B. tabaci* or *E. hayati* in combination has not been studied. In the current study, three formulations comprising varying combinations of mustard oil and the surfactants monoethanolamine (MEA), lauryl glucoside, laureth carboxylate, and cellosolve acetate were assessed for efficacy against the different developmental stages of *B. tabaci* in a laboratory. Furthermore, an assessment was conducted on the possible adverse effects of the three formulations on the parasitoid *E. hayati*. The outcomes of this investigation were categorized based on the toxicity rank established by the International Organization for Biological Control.

## 2. Results

### 2.1. Toxicity Effects of the EO Formulations against the Eggs, Nymphal, and Adult Stages of B. tabaci

**Figure 1:**
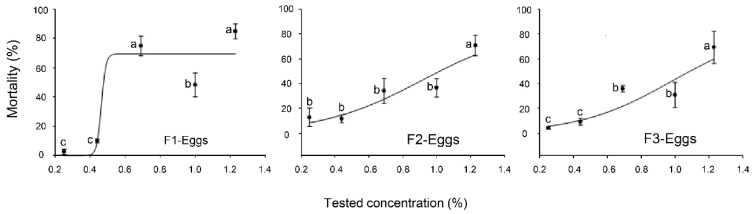
Mortality (%) of three formulations (F1, F2, and F3) against eggs of *Bemisia tabaci* at different concentrations. The data are presented as means ± SE of four replicates. Different letters at each point indicate that there are statistically significant differences (*p* < 0.05) between the concentrations of each formulation that were tested.

All three formulations showed different mortality rates against the eggs of *B. tabaci* (Figure 1). At a concentration of 1.23%, the highest mortality rates of F1, F2, and F3 were 85%, 70.8%, and 69.2%, respectively (Figure 1).

All the tested concentrations of F1 exhibited significant mortality rates compared to the control for the eggs of *B. tabaci* (*p* < 0.05) (Figure 1). On the other hand, only the higher concentration of F2 (1.23%) revealed significant mortality rates in comparison to the control for the eggs of *B. tabaci* (*p* < 0.05) (Figure 1). However, when compared to the other formulations which had been tested, F3 had the lowest mortality rates for the eggs (Figure 1). The lethal concentrations (LC_50_ and LC_90_) of the tested formulations (F1, F2, and F3) against the eggs of SLW are presented in Table 1. The LC_50_ value of F1 (0.73%) was significantly lower than that of F2 and F3 (1.02 and 1.05%, respectively), as shown by the non-overlap of the 95% confidence intervals (Table 1).

**Figure 2:**
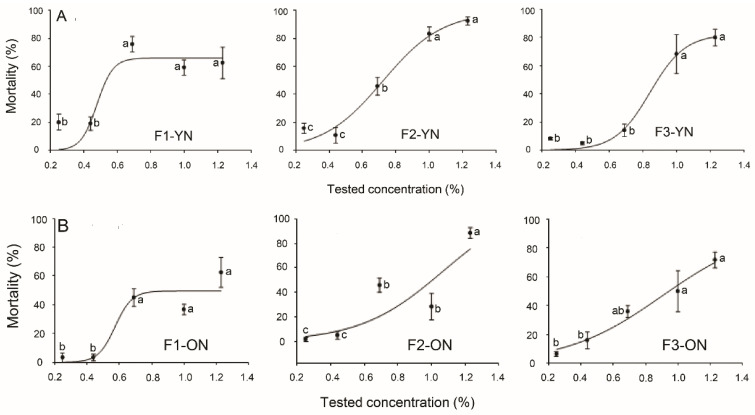
Mortality (%) of three formulations (F1, F2, and F3) against younger ((**A**)-first and second instars) and older nymphs ((**B**)-third and fourth instars) of *Bemisia tabaci* at different concentrations. The data are presented as means ± SE of four replicates. Different letters at each point indicate that there are statistically significant differences (*p* < 0.05) between the concentrations of each formulation that were tested.

The mortality (%) resulting from the toxicity of the three formulations against the young (in their first and second instars) and older (in their third and fourth instars) nymphs of SLW are presented in Figure 2. The highest mortality (%) of the young nymphs of SLW was recorded for F1 as 75.83%, whereas the highest mortality rates (%) for F2 and F3 were 92.5 and 80%, respectively, at a concentration of 1.23% (Figure 2A). The LC_50_ values for the young nymphs of SLW for F1, F2, and F3 were 0.69, 0.65, and 0.88%, respectively (Table 1). On the other hand, the highest mortality rates (%) of the older nymphs (third and fourth instars) were recorded as 62.5, 88.33, 71.67%, respectively, at 1.23% concentrations (Figure 2B). There were no significant differences between the LC_50_ values of the formulations for the older nymphs (Table 1). Their LC_50_ values were 1.03%, 0.91%, and 0.9% for F1, F2, and F3, respectively (Table 1).

**Figure 3:**
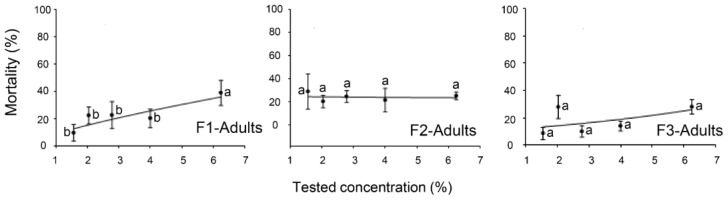
Mortality (%) of three formulations (F1, F2, and F3) against adults of *Bemisia tabaci* at different concentrations. The data are presented as means ± SE of four replicates. Different letters at each point indicate that there are statistically significant differences (*p* < 0.05) between the concentrations of each formulation that were tested.

Figure 3 presents the results of mortality (%) due to the formulations at different concentrations (1.56%, 2.04%, 2.78%, 4%, and 6.25%) against the adults of SLW. Adult mortalities were low at all the tested concentrations. The mortality was always less than 30% except for F1 at a 6.25% concentration, which showed a 38.54% adult mortality (Figure 3). Therefore, the LC_50_ and LC_90_ could not be calculated for the adult stages.

### 2.2. Toxicity of EOs Formulations against the Parasitoid E. hayati

**Figure 4:**
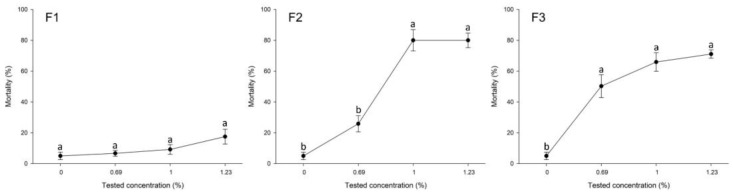
Mortality (%) of plant essential oil formulations at different tested concentrations against SLW parasitoid, *Eretmocerus hayati*. The data are presented as means ± SE of eight replicates. Different letters at each point indicate that there are statistically significant differences (*p* < 0.05) between the concentrations of each formulation that were tested.

According to our results, F1 had the lowest lethal impact on the parasitoid compared to the other two formulations. At the highest concentration rate (1.23%), it resulted in 17.5% mortality, classed as harmless (<30% mortality) according to the IOBC guidelines [44]. F2 resulted in 80% mortality at the highest tested concentrations (1% and 1.23%), classified as moderately harmful (80–99% mortality). The three tested concentrations of F3 resulted in mortality between 53.3% and 70% (classified as slightly harmful, 30–79% mortality) (Figure 4).

For the F1, there was no significant effect of the treatment on mortality (F = 2.24, df = 3.28, *p* > 0.05) (Figure 4). However, there was a significant effect of the treatment on the mortality of the parasitoids for F2 (F = 43.77, df = 3.28, *p* < 0.0001) and F3 (F = 29.92, df = 3.28, *p* < 0.0001) (Figure 4).

## 3. Materials and Methods

### 3.1. Insects and Plants

A laboratory strain of *B. tabaci* MEAM1 were initially obtained from a colony reared in the Department of Agriculture and Fisheries (DAF) laboratories at the Leslie Research Centre in Toowoomba (Rockville, QLD, Australia). The whiteflies were reared on cotton plants (*Gossypium hirsutum*, variety Sicot 71RRF) in 45 × 45 × 45 cm cages in an insectary at the University of Queensland, Gatton Campus (Gatton, QLD, Australia), maintained at 27 ± 2 °C, RH 60 ± 10%, and a 14:10 (light:dark) photoperiod. The population had not been exposed to any pesticides since the time of collection.

The wasp parasitoids were obtained from Bugs for Bugs Pty Ltd. (Toowoomba, QLD, Australia). The wasps were provided in small, plastic vials plugged with cotton. They were released into cages (45 × 45 × 45 cm) containing tomato seedlings infested with mixed developmental stages of SLW and maintained in an insectary at the University of Queensland, Gatton Campus, maintained at 27 ± 2 °C, RH 60 ± 10%, and a 14:10 (light:dark) photoperiod.

Cotton seeds were obtained from the DAF Leslie Research Centre. Tomato seedlings, *Lycopersicon esculentum* Mill. (Solanaceae), variety Grosse Lisse, were obtained from a local nursery in Gatton. Two seedlings were transplanted into 1.5 L plastic pots using potting media. The media consisted of composted pine bark and woodchips. The seedlings were grown in a greenhouse and were watered regularly using an automatic watering system.

### 3.2. Essential Oil Formulations

In the current study, three formulations (referred to below as F1, F2, and F3) were assessed (Table 2).

Lauryl glucoside is derived from lauryl alcohol (from coconut or palm) and glucose (from corn or potato). In addition, lauryl glucoside is a plant-based surfactant and used in cleansing agents and also personal care products [45]. The MW-100 emulsifier is a single component vegetable oil emulsifier also used in mineral oil-based formulations. The mustard plant is classified as an annual plant and is a member of the *Brassicaceae* plant family [46]. Mustard oil is extracted from the seeds of the *Brassicaceae* plant family [46]. The primary constituent of mustard oil is isothiocyanate. Allyl isothiocyanate is found in high concentrations (71.06%) in mustard oil [47]. Monoethanolamine is a naturally occurring organic chemical compound used in a wide range of applications, including acid gas purification, surfactants for detergents and personal care products, and intermediates for agrochemical use [48]. Each formulation was tested at five concentrations (0.25%, 0.44%, 0.69%, 1%, and 1.23%) for its effects on the eggs and nymphal stages of *B. tabaci* and compared with water as a control. For the adults, concentrations of 1.56%, 2.04%, 2.78%, 4%, and 6.25% for each formulation were tested. In addition, three concentrations for each formulation (0.69%, 1%, and 1.23%) were used for treating the parasitoid, *E. hayati*. The concentrations used were established in preliminary trials. The concentrations were generated as a percentage in a 100 mL water solution. All the formulations were supplied by BioAust Pty Ltd. (Jimboomba, QLD, Australia).

### 3.3. Toxicity of the EO Formulations against the Eggs, Nymphal, and Adult Stages of B. tabaci

A day before the experiment, tomato leaves were removed from the seedlings with a razor blade. The leaves were placed in 20 mL plastic tubes filled with water. For the egg test, 10 male and 10 female adults of SLW were introduced into clip cages (2 cm in diameter) where they deposited eggs. The adults were removed after 24 h. Thirty eggs per leaflet were counted, and a mark was put beside each egg using a waterproof pen. Each leaflet was counted as a replicate, and there were four replicates per test. Three days later (three-day old eggs), the leaves were sprayed with the EO formulation or with water as a control. The sprayed leaves were left to dry, then returned to the 20 mL plastic tube filled with water for 7 days until egg hatching was completed.

For the nymphal mortality test, 15 adults were aspirated and introduced to each leaf in a clip cage for 24 h where they deposited eggs, as described for the egg test. Treatments were applied ten days after adult removal for younger nymphs (in their first and second instars) and eighteen days for older nymphs (in their third and fourth instars). The nymphs were counted and marked under a dissecting microscope with a waterproof pen. The leaves were sprayed with the prepared solutions, left to dry, and then returned to the 20 mL plastic tube filled with water. Three days after treatment, the mortality percentages were calculated. Brown nymphs that were shrunken and dried were counted as dead.

For the adult mortality test, 20–30 adults (males and females) were aspirated from the tomato plants and introduced into a clip cage. A leaflet was sprayed thoroughly to run-off and was immediately inserted into the clip cage containing the adults. For the adults, the formulations were tested at higher concentrations (1.56%, 2.04%, 2.78%, 4%, and 6.25%) as preliminary tests had shown that the concentrations used against the immature life stages were not effective on the adults. There were four replicates (each leaflet in one clip cage was considered as one replicate) for each concentration of each formulation. Water was used as a control. The mortality percentages were calculated 24 h after the adults were first exposed to a sprayed leaflet. The adults were counted as dead when they remained immobile after being touched with a fine paintbrush. All the treated insects were maintained at 27 ± 2 °C, RH 60 ± 10%, and a 14:10 (light:dark) photoperiod.

### 3.4. Toxicity of EO Formulations against the Parasitoid E. hayati

The parasitoid wasps were released and allowed to parasitize the SLW nymphs. After two weeks, the tomato leaves were removed from the seedlings and the parasitized nymphs were identified. The parasitized nymphs were exposed to the treatments using a glass slide bioassay [49]. Three droplets (10 µL each) of each of the prepared diluted solutions (0.69%, 1%, and 1.23%) of F1, F2, and F3 and the water control were placed on a glass slide. The parasitized nymphs were carefully removed from the leaf and five of them were placed into each droplet. The slides were left to dry in a laminar hood for 30 min and then placed into Petri dishes lined with wet filter paper, with no lid in place. The slides were held in cages under laboratory conditions (24 ± 2 °C and a 14:10 light:dark photoperiod). There were eight replicates for each formulation, with each replicate comprising one slide bearing 15 parasitized nymphs. Mortality was assessed 48 h after treatment. Shrivelled and/or discoloured nymphs were considered dead.

### 3.5. Statistical Analysis

The mortality percentages of *B. tabaci* and *E. hayati* for each formulation were calculated and subjected to a one-way analysis of variance (ANOVA). Subsequently, Tukey’s test was used to differentiate between the means of the treatments (Minitab 17).

Additionally, a probit analysis was conducted to determine the LC_50_ and LC_90_ values for the formulations when tested against the eggs and nymphal stages of *B. tabaci*.

## 4. Discussion

From the above results, it can be recognised that the three formulations were effective against all the developmental stages of *B. tabaci* under laboratory conditions, although the efficacy of each formulation varied depending on life stage. F1 was most effective against the eggs of *B. tabaci*, whereas F2 showed the highest toxicity against the young and old nymphs of *B. tabaci* at the highest tested concentration (1.23%). In comparison, all the formulations resulted in a lower mortality of adults of *B. tabaci*, namely, less than 40% at the highest concentration (1.23%) of each formulation.

In our study, we used mustard EO (20%) in each formulation. Prior research demonstrated that mustard EO showed strong insecticidal activity against the larvae of *Cydia pomonella* L. (Lepidoptera: Tortricidae) compared to the *Spodoptera exigua* Hübner (Lepidoptera: Noctuidae) and *Dendrolimus pini* L. (Lepidoptera: Lasiocampidae), with LC_50_ values of 0.42, 11.66, and 11.74 mg/mL, respectively [37]. However, until now, the toxicity of mustard EO had not been studied against *B. tabaci*. Several studies have evaluated essential oils against the eggs, nymphs, and adults of *B. tabaci*. Yang et al. (2010) [50] observed that the essential oil extracted from garden thyme, *Thymus vulgaris* L., at 0.5% reduced the survival rate of *B. tabaci* by 73.4%, 79.0%, and 58.2% after the treatment of eggs, first nymphal instar, and fourth nymphal instars, respectively. In addition, Al-mazra’awi and Ateyyat (2009) [51] evaluated nine plant extracts against eggs, nymphs, and adults of *B. tabaci*, finding that efficacy varied according to the life stage. The highest efficacy was obtained for the second nymphal instar (80, 77, and 67% mortality for extracts of *Peganum harmala* L., *Anthemis palaestina*, and *Ruta chalepensis* L., respectively), compared to more than 50% mortality for the third instar nymphs treated with extracts of *R. chalepensis* and *Alkanna strigosa* Boiss. & Hohen. The percentage of unhatched eggs ranged between 0 and 33%, and the tested extracts were generally ineffective against the adult stage. However, Kim et al. (2011) [52] demonstrated the high mortality effects of nine essential oil formulations against adult females of *B. tabaci* using a spray bioassay. Mostafiz et al. (2018) [27] also reported that a volatile organic compound, methyl benzoate, exhibited insecticidal activity against the different life stages of *B. tabaci,* although the LC_50_ values varied between the eggs, nymphs, and adults of *B. tabaci*. Therefore, our results were broadly similar to those reported for other studies on the effectiveness of essential oil formulations against the immature stages of *B. tabaci*.

Several studies have evaluated the impact of plant extracts on parasitoids and found a range of effects. For example, Simmons and Shaaban (2011) [53] found that the biorational insecticides jojoba oil, Biovar, and Neemix had the least effect on the abundance of natural enemies, including *Eretmocerus* spp. (Hymenoptera: Aphelinidae), in comparison to other insecticides during a 14-day evaluation period. Extracts of *R. chalepensis*, *Peganum harmala* L., and *A. strigose* were found to be effective against *B. tabaci* and have minimal effects on its parasitoid *Eretmocerus mundus* Mercet (Hymenoptera: Aphelinidae) [51]. Conversely, Kumar et al. (2008) [54] showed that neem oil caused high mortality rates when it was sprayed on SLW nymphs parasitized with *Eretmocerus warrae* (Hymenoptera: Aphelinidae). However, in another study, methyl benzoate was shown not to have strong lethal and sublethal effects on the two generalist predators *Nesidiocoris tenuis* Reuter (Hemiptera: Miridae) and *Orius laevigatus* Fieber (Hemiptera: Anthocoridae) [33,34]. In the current study, F1 was identified as not harmful to the parasitoid *E. hayati*. However, F2 and F3 were classified as moderately and slightly harmful to *E. hayati*, respectively. The toxicity of a pesticide depends on different factors such as the target species’ body size, developmental stage (egg, larva, or adult), and behaviour and the pesticide’s mode of action, dosage, and method of application [55,56,57]. Further investigation is required to better understand the observed differences in susceptibility of *B. tabaci* and *E. hayati* to the tested formulations in the current study. Nevertheless, it is necessary to adjust the amounts of the components in the formulation in order to avoid phytotoxicity and maximize the toxicity of the formulations. Furthermore, it is essential to undertake further research in order to evaluate the efficacy of formulation 1 when applied to the young stages of the parasitoid *E. hayati*, both in controlled laboratory settings and in field trials.

In addition, the efficacy of EOs has been assessed in field settings to determine their performance under uncontrolled circumstances. As an example, the EO from *Ocimum basilicum* L. has shown a lot of promise as a biopesticide against *Helicoverpa armigera* (Hubner) (Lepidoptera: Noctuidae) in the field [58]. In another study, it was shown that neem-derivative compounds, particularly Azaridichtin, exhibited efficacy as insecticides against *H. armigera* in both laboratory and field studies [59]. Furthermore, it is important to conduct further research in order to explore new formulations and application techniques that have the potential to enhance the efficacy and durability of essential oils in practical scenarios.

The use of IPM has been reported as a cost-effective strategy that successfully reduces crop losses [60]. Plant pesticides are considered to be more cost-effective and sustainable in comparison to synthetic pesticides [61,62]. Hence, biopesticides have attracted significant attention due to their capacity to specifically target pests, their effectiveness, their ability to degrade naturally, their environmental safety, and their applicability in IPM programs [63]. Biopesticides show great potential as viable solutions for reducing environmental contamination caused by synthetic chemical pesticides.

The adequate production of food and fibre has always been a significant difficulty in human societies. In general, it can be seen that about one-fifth of the global food supply is yearly compromised by the adverse effects of insect and mite pests, diseases, and weeds. Environmental concerns, food safety, insect resistance, human and animal health, and sustainable practices were the primary motivating factors for the majority of researchers to switch their focus from chemically based pest control to biologically and ecologically based pest management. In the context of promoting the advancement of sustainable agricultural programs, it is plausible to consider that the use of environmentally friendly organic compounds and their corresponding formulations may provide a more favourable alternative. The findings of our research will contribute to the advancement of sustainable agriculture.

## 5. Conclusions

Three plant essential oil formulations were tested in this study for the first time against a major agricultural pest, the silverleaf whitefly. All three formulations were effective against the immature stages (eggs and nymphs) at the highest concentration tested but were less effective against the adult stage. In comparison, formulation 1 was found to be harmless to the parasitoid *E. hayati* even at the highest concentration tested. Therefore, formulation 1 could play a part in IPM programs for SLW targeting of the younger life stages. As formulation 1 was less effective against older nymphs and ineffective against adults, any IPM program including this formulation should also include, for example, a repellent or oviposition deterrent to target the adult whitefly. Further experiments are needed to determine the combination effects of formulation 1 and the parasitoid *E. hayati* in greenhouse and open field conditions. Moreover, it is essential to conduct more research to evaluate the effectiveness of the formulations in addressing other agricultural insect pests.

## Figures and Tables

**Table 1:** The LC_50_ and LC_90_ values for the developmental stages of *Bemisia tabaci* treated with three essential oil formulations under laboratory conditions.

Formulations	SLW Stage	n	LC_50_		LC_90_		*p*
			(%) (±SE)	95% CI	(%) (±SE)	95% CI	
F1	Egg	600	0.73 (±0.026)	0.684–0.789	1.59 (±0.117)	1.405–1.885	<0.001
F2	Egg	599	1.02 (±0.062)	0.913–1.169	3.43 (±0.601)	2.580–5.299	<0.001
F3	Egg	600	1.05 (±0.052)	0.958–1.168	2.69 (±0.348)	2.172–3.685	<0.001
F1	YN	600	0.69 (±0.042)	0.608–0.778	3.15 (±0.60)	2.317–5.061	<0.001
F2	YN	600	0.65 (±0.023)	0.608–0.698	1.36 (±0.086)	1.213–1.565	<0.001
F3	YN	600	0.88 (±0.028)	0.830–0.942	1.68 (±0.114)	1.500–1.971	<0.001
F1	ON	600	1.03 (±0.048)	0.944–1.139	2.53 (±0.305)	2.076–3.390	<0.001
F2	ON	600	0.91 (±0.031)	0.854–0.976	1.81 (±0.136)	1.589–2.152	<0.001
F3	ON	589	0.90 (±0.041)	0.829–0.994	2.40 (±0.285)	1.968–3.187	<0.001

n-total number of tested individuals for each formulation; LC50 and LC90 values are in (%). SE—standard error; CI—confidence interval; YN—young nymphs (in the first and second instars); ON—old nymphs (in the third and fourth instars).

**Table 2:** The components of the three essential oil formulations and their percentages.

Formulation 1	Formulation 2	Formulation 3	%
Lauryl glucoside	Laureth carboxylate	Lauryl glucoside	20
MW 100 emulsifier	MW 100 emulsifier	MW 100 emulsifier	40
Mustard oil	Mustard oil	Mustard oil	20
Cellosolve acetate	Monoethanolamine	Monoethanolamine	20

## Data Availability

Data are contained within the article.
